# Tear-derived exosomal biomarkers of Graves’ ophthalmopathy

**DOI:** 10.3389/fimmu.2022.1088606

**Published:** 2022-12-06

**Authors:** Ting-Ting Shi, Ru-Xuan Zhao, Zhong Xin, Zhi-Jia Hou, Hua Wang, Rong-Rong Xie, Dong-Mei Li, Jin-Kui Yang

**Affiliations:** ^1^ Department of Endocrinology, Beijing Tongren Hospital, Capital Medical University, Beijing, China; ^2^ Beijing Key Laboratory of Diabetes Research and Care, Beijing Tongren Hospital, Capital Medical University, Beijing, China; ^3^ Department of Ophthalmology, Beijing Tongren Hospital, Capital Medical University, Beijing, China; ^4^ Department of Emergency, Beijing Tongren Hospital, Capital Medical University, Beijing, China

**Keywords:** Graves’ ophthalmopathy, biomarker, exosome, tear, Caspase-3, APOA-IV

## Abstract

Graves’ ophthalmopathy (GO), the most frequent extrathyroidal manifestation of Graves’ disease (GD), can lead to a significant decline in the quality of life in patients. Exosomes, which contain proteins, lipids and DNA, play important roles in the pathological processes of various diseases. However, their roles in Graves’ ophthalmopathy are still unclear. We aimed to isolate exosomes and analyze the different exosomal proteins. Tear fluids were collected from twenty-four GO patients, twenty-four GD patients and sixteen control subjects. The numbers of tear exosomes were assayed using nanoparticle tracking analysis. A Luminex 200 kit and ELISA kit were used to confirm the different cytokine concentrations in serum. Extraocular muscle from GO patients and controls was extracted, and western blotting was used to assay the levels of Caspase-3 and complement C4A. Our study demonstrated that the number of tear exosomes differ from GD patients and control. The expression levels of cytokines, including IL-1 and IL-18, were significantly increased in the tear exosomes and serum from GO patients compared with GD patients and controls. The levels of the exosomal proteins Caspase-3, complement C4A and APOA-IV were significantly increased in GO patients compared to GD patients and controls. Orbital fibroblasts from GO patients showed significantly higher levels of Caspase-3 and complement C4A than those from controls. The levels of serum APOA-IV in GO patients were significantly higher than those in GD patients and controls. Specific proteins showed elevated expression in tear exosomes from GO patients, indicating that they may play important roles in GO pathogenesis.

## Introduction

Graves’ ophthalmopathy (GO) is the most frequent extrathyroidal manifestation of Graves’ disease (GD) (1). GO is also an organ-specific autoimmune disease. Eyelid swelling, eyelid retraction, proptosis, excessive tearing, periorbital edema, and diplopia are the characteristic clinical signs of Graves’ ophthalmopathy (2). Patients with optic nerve compression suffer from vision loss, which seriously affects the quality of life of patients. Pathophysiologically, the disease is characterized by orbital inflammation, ocular tissue expansion, remodeling and ultimately fibrosis (3, 4). It is essential to diagnose active and severe GO at the early stage to prevent further progression.

In GO patients, the evaporation of tears may be caused by long-term ocular exposure because of eyelid retraction and proptosis (5). Studies have shown that lacrimal glands in GO patients are involved in autoimmune responses, leading to changes in the composition of tears (6). Therefore, changes in tears and lacrimal glands might be considered risk factors for progression in GO patients (7). The biological information in tear fluid is helpful for studying the mechanisms of GO. Recently, proteomic analysis of the tear fluids of patients with GO was employed to identify potential biomarkers for disease activity and progression. Eckstein et al. reported that thyroid-stimulating hormone receptor (TSH-R) autoantibodies and inflammatory cytokines in tears are important components promoting the progression of GO (8). Evidence has shown that the levels of tumor necrosis factor (TNF-α), interleukin (IL)-1, IL-6, IL-13, and IL-18 are increased in GO patients (9).

Exosomes are extracellular membranous vesicles with diameters of 30-150 nm that can be found in numerous body fluids, such as plasma, urine, saliva, breast milk and tears. Exosomes play important roles in mediating intra- and extracellular signaling and modulating immune responses in diseases (10). Fitzgerald et al. has demonstrated that IL-17 and TNF-α were found in higher levels in exosomes in most systems. They also showed that IL-18 and TNF-α were preferentially encapsulated in exosomes in most systems (11). However, the roles of these proteins in GO are unclear (9).

In this study, we aimed to investigate specific proteins or cytokines in tear exosomes that are differentially expressed in individuals with GO compared with individuals with GD and healthy controls.

## Materials and methods

### Subjects

In the current study, label-fee proteomics was performed to determine the marker exosomes using the tear samples of GO patients and controls. Our study recruited 24 active and severe GO patients with hyperthyroidism at the time of assessment (9 males, 15 females), 24 newly diagnosed GD patients (10 males, 14 females) and 16 healthy controls (5 males, 11 females) between January 2018 and January 2019. This research was carried out at the Department of Endocrinology, Beijing Tongren Hospital, Capital Medical University, Beijing, China. Thyroid function in the GD group was comparable to that in the active GO group. All active GO and GD patients received treatment only with antithyroid drugs. The active GO patients in this study had not received any treatment, such as steroids, thyroid surgery or radiotherapy. GO patients were diagnosed by the guidelines of The European Group on Graves’ ophthalmopathy (EUGOGO) (12). Disease with a clinical activity score (CAS) ≥ 3 was defined as active GO, and disease with a NOSPECS score ≥ IV was defined as severe GO. Patients with GO who had a history of orbital trauma, ocular tumors, or any other lacrimal gland diseases were excluded. The characteristics and clinical data of the three groups of subjects are summarized in **Tables 1**, **2**.

**Table 1:** Demographic data of patients with Graves’ ophthalmopathy, patients with Graves’ disease and healthy controls.

	GO	GD	N
Famale(%)	15(62.5)	14(58.3)	11(68.7)
Male(%)	9(37.5)	10(41.7)	5(31.3)
Smokers(%)	10(41.7)	5(20.8)	3(18.8)
Age,years	45.2 ± 10.1	40.3 ± 8.9	40.4 ± 9.6
CAS	3.6 ± 0.3	NS	NS
NOSPECS(%)	IV 22(91.7), V 2(8.3)	NS	NS

**Table 2:** Clinical data of patients with Graves’ ophthalmopathy, patients with Graves’ disease and healthy controls.

	GO	GD	N
T3(pmol/L)	3.17(0.95,5.92)	3.51(1.23,6.53)	1.28(0.62,2.67)^b,c^
T4(pmol/L)	142.28(68.30,196.60)	159.02(28.80,274.10)	67.86(58.92,130.59)^b,c^
FT3(pmol/L)	9.18(3.11,18.95)	11.26(3.80,27.62)	4.96(3.35,6.29)^b,c^
FT4(pmol/L)	25.83(13.06,40.91)	27.09(12.71,52.08)	13.4(9.72,17.41)^b,c^
TSH(uIU/ml)	0.02(0.00,0.11)	0.02(0.00,0.14)	2.1(1.06,3.60)^b,c^
TPOAb(IU/ml)	70.56(10.33,150.94)	82.49(9.74,140.81)	20.83(5.33,35.13)^b,c^
TGAb(IU/ml)	261.54(10.10,1231.00)	304.09(10.82,1402.00)	48.17(3.09,100.38)^b,c^
TRAb(IU/L)	11.40(2.38,35.75)	8.78(1.51,24.96)^a^	0.38(0.3,0.93)^b,c^
ALT(U/L)	16.13(8,29)	20.57(8,36)	21.80(9,37)
AST(U/L)	18.6(12,29)	19.68(12,35)	21.05(14,40)
BUN(mmol/L)	4.74(2.6,6.5)	4.75(2.6,9.1)	5.52(2.5,9.6)
CREA(umol/L)	61.67(50,70.6)	63.74(50,94)	68.01(48,93.6)
Tear volume(mm/eye)	14.5(5,30)	11.6(5.5,30)	12.3(5,30)

The values are the mean (SD), median (range), or n (%). Comparison between any two groups was performed by Mann–Whitney U test. ^a^P < 0.05 between patients with Graves’ ophthalmopathy and Graves’ disease; ^bc^P < 0.001 healthy controls compared with patients with Graves’ ophthalmopathy or Graves’ disease. CAS, clinical activity score; NOSPECS, no signs or symptoms, only signs, soft tissue, proptosis, extraocular muscle, cornea, sight loss; TSH, thyroid-stimulating hormone; TPOAb, thyroperoxidase antibody; TGAb, antithyroglobulin antibody; TRAb, thyrotropin receptor antibody. Normal ranges: T3 0.92-2.79nmol/L, T4 57.9-140.3nmol/L, FT3 3.5-6.5pmol/L, FT4 11.5-22.7pmol/L, TSH 0.55-4.78uIU/ml, TPOAb 0-40 IU/ml, TGAb 0-115IU/ml, TRAb 0-1.75IU/L.

This study was approved by the Ethics Committee of Beijing Tongren Hospital, Capital Medical University. The consent of all enrolled subjects was obtained after the study process was fully explained.

### Tear collection

Tear samples were collected *via* the Schirmer test using Schirmer strips (Tianjing Jingming New Technological Development Co., Ltd., China) according to a previous study (13). The strips were inserted for 5 minutes into the inferior conjunctiva to 1/3 the distance from the lateral canthus, while patients kept their eyes closed. During this procedure, touching of the eyelid or ocular surface was avoided to minimize the chance of contamination or eye irritation. After collection, these strips were immediately frozen at −80°C.

### Exosome extraction

**Figure 1:**
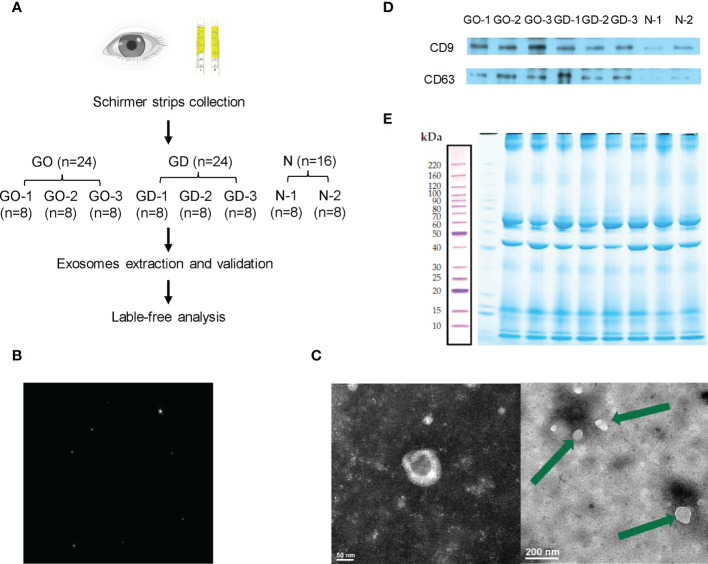
Workflow and preparation before LC−MS/MS analysis. **(A)** Experimental workflow of this study. **(B)** Representative nanoparticle tracking analysis of tear exosomes. **(C)** Representative transmission electron microscopy images of tear exosomes. The scale bar on the left represents 50 nm, and the scale bar on the right represents 200 nm. **(D)** Expression of CD9 and CD63 in exosomes of active GO patients, GD patients and healthy controls. **(E)** Exosomal protein presented in SDS-PAGE with Coomassie Brilliant Blue.

Tear exosomes were extracted using an exoEasy Maxi Kit (Qiagen, USA) according to the manufacturer’s protocol. Eight Schirmer strips from the same group were combined into one pooled group for exosome extraction. Thus, 24 strips from active GO patients were divided into 3 subgroups. Twenty-four strips from GD patients were divided into 3 subgroups. Sixteen strips from healthy subjects were divided into 2 subgroups (**Figure 1A**). XBP buffer was added to each tear sample before it was coupled to an exoEasy membrane affinity spin column. Wash buffer was added to the tear exosomes, and buffer XE was used for elution.

### Nanoparticle tracking analysis

Fifty microliters of exosome suspension was rediluted with XE buffer at 1:1000 and then placed on a Zetaview nanoparticle tracking analyzer (Particle Metrix, Germany) for analysis and detection.

### Transmission electron microscopy

The exosome suspension was centrifuged at 100,000 × *g* for 70 minutes using a Beckman analytical ultracentrifuge. Then, the supernatant was discarded, and the previous step was repeated. The precipitated sample was resuspended and dropped onto a copper mesh for 30 minutes. Later, 2% phosphotungstic acid was used to counterstain the exosomes. Utilizing a JEM-1400 transmission electron microscope, the stained samples were analyzed (JEOL, Japan).

### Western blot analysis

Exosomes were lysed with Laemmli buffer, and retrobulbar adipose tissue was lysed with RIPA buffer to extract total protein. Sodium dodecyl sulphate polyacrylamide gel electrophoresis (SDS-PAGE) was used to electrophorese the exosomal proteins. After that, a polyvinylidene fluoride (PVDF) membrane was used to transfer the proteins in the gel. The PVDF membrane was blocked using 5% skim milk. The membranes were incubated with primary antibodies against CD9 (Cell Signaling Technology, USA), CD63 (Cell Signaling Technology, USA), Caspase-3 (Cell Signaling Technology, USA), complement C4A (Abcam, UK) and GAPDH (Cell Signaling Technology, USA). The signals of the blots were generated with Lumi-Light Western Blotting Substrate (Roche, USA) and visualized with a ChemiDoc MP Imaging System (Bio-Rad, USA).

### Exosome digestion

Exosomes were lysed using 8 M urea, 50 mM NH_4_HCO_3_, 50 mM iodoacetamide (IAA), and 1× protease inhibitor with ultrasound on ice. Then, the mixture was centrifuged at 13,300 rpm for 5 minutes at 4°C. The cellular supernatant was subjected to SDS−PAGE and stained with Coomassie Brilliant Blue. For protein trypsinization, exosomal protein was added to 1 M dithiothreitol (DTT) and 1 M IAA in the dark. One hour later, the mixture was transferred to ultrafiltration spin columns and successively filtered with UA (8 M urea, 100 mM Tris-HCl, pH 8.0) and 50 mM NH_4_HCO_3_. Finally, trypsin was added to the ultrafiltration tube, and the tube was incubated at 37°C for 12 hours. The digested mixtures were stored at -80°C for subsequent MS analysis.

### LC−MS/MS analysis

Peptide mixtures were subjected to nano-liquid chromatography coupled with MS to achieve protein identification. An Agilent 1100 quaternary HPLC (Agilent, EASY-nLC1000, USA) and a Q-Exactive mass spectrometer (Thermo Finnigan, Germany) were included in the LC−MS/MS system that was used to perform the mass analyses. Samples were desalted using an RP trap column and separated using a C18 reverse-phase column. HPLC-grade water with 0.1% formic acid (FA) formed phase A, and 84% HPLC-grade acetonitrile with 0.1% FA formed phase B. A scan of the MS survey scans included one full MS scan and ten analyzed MS/MS events. To reduce the technical variation, the analyses were repeated 3 times.

### Bioinformatics analysis

The Mascot 2.1 program was used to search the fragmentation spectra for protein identification. MaxQuant MS Analysis Software was used to analyze the fold changes of the differentially expressed proteins in the groups. AgBase Version 2.0 is an accessible resource for identifying proteins based on their cellular components, molecular functions and biological processes and in compliance with Gene Ontology standards (14). Metascape (https://metascape.org) was used to perform the signaling pathway analysis (15).

### Inflammatory cytokine measurements

Cytokines were measured using Human Cytokine Screening 48-Plex Services according to the manufacturer’s instructions by Novogene CoLtd (Beijing, China). The plate was read on a Luminex MAGPIX instrument (Luminex). Data acquisition and analysis was conducted using the Luminex xPONENT software.

### ELISA measurements

The serum apolipoprotein A-IV (APOA-IV) content was quantified in active GO patients with hyperthyroidism (n=24), matched GD patients with hyperthyroidism (n=24) and healthy controls (n=24). A human APOA-IV enzyme-linked immunosorbent assay kit (Cusabio Biotech, China) with a detection range of 0.041-30 ng/ml was used. All procedures were carried out according to the manufacturer’s instructions. Serum protein concentrations were detected at a wavelength of 450 nm using the standard curve with a microplate reader (Thermo Fisher Scientific, USA).

### Orbital tissue acquisition

Orbital tissues were obtained from three patients with GO in an active stage (clinical activity score (CAS) ≥ 3/7) who underwent orbital decompression surgery. In addition, orbital tissues were obtained from three controls that underwent orbital surgery for other reasons, as described previously.

### Statistical analysis

Statistical analysis was performed by SPSS v25.0. The mean ± SD is used to describe continuous variables. Normally and nonnormally distributed data were analyzed with Student’s t tests and Mann−Whitney U tests. A two-tailed p < 0.05 was considered the significance threshold for this study.

## Results

### Demographic and clinical data

A demographic and clinical overview of the three groups, including 24 active and severe GO patients, 24 newly diagnosed GD patients and 16 healthy controls, is summarized in **Table 1** and **Table 2**. No distinct differences were found in age or sex among the three groups. There were also no differences in smokers in the three groups, and the tear amounts of the three groups were almost the same. Healthy controls had normal levels of thyroid function and thyroid-related antibodies, including thyroperoxidase antibody (TPOAb), antithyroglobulin antibody (TGAb) and thyrotropin receptor antibody (TRAb), which were different from the levels in the GO and GD patients (all p < 0.001). TRAb values were different between active and severe GO patients and GD patients (p=0.023), where GO patients had higher serum TRAb levels of 11.4 IU/L. Liver and kidney function of these patients were listed in the **Table 2**.

### Validation of exosomes

The pipeline of the study is shown in **Figure 1A**. After collecting and grouping all the tear samples, exosomes were extracted and verified. The exosomes of tears were verified by nanoparticle tracking analysis, transmission electron microscopy and western blotting. The exosomes detected by nanoparticle tracking analysis were 125.6 nm in diameter (**Figure 1B**). Transmission electron microscopy showed that the exosomes were irregular or spherical vesicles ranging from 30 nm to 200 nm (**Figure 1C**). Exosomal biomarkers, such as CD9 and CD63, were further detected by western blotting of samples from active GO patients, GD patients and healthy controls (**Figure 1D**).

### Protein identification

Exosomal protein was extracted, subjected to SDS−PAGE and stained with Coomassie Brilliant Blue (**Figure 1E**). The results showed that effective separation was achieved in the molecular weight range of 10-220 kDa and that the total protein was not degraded. Overall, the proteins in the 8 samples had similar banding patterns, and the total amount of protein was sufficient to complete the subsequent experiments.

### Proteomic analysis

**Figure 2:**
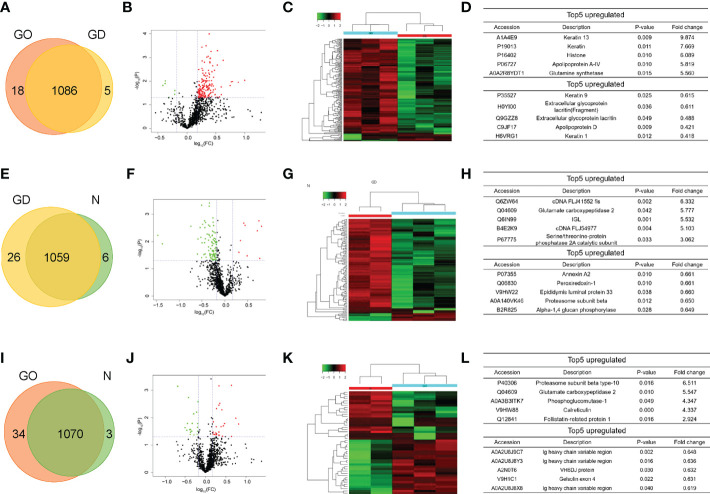
Pairwise comparison of the differentially expressed proteins in exosomes among the three groups. **(A, E, I)** Venn diagram comparing the number of identified proteins between GO and GD, GD and controls, GO and controls. **(B, F, J)** Volcano plots showing the differentially expressed proteins in exosomes between the GO group and the GD group, between the GD group and the healthy control group, and between the GO group and the healthy control group. A fold change of 1.5 and a p < 0.05 were used as the cutoff points. Upregulated proteins are shown in red, and downregulated proteins are shown in green. **(C, G, K)** Clustering heatmaps of differentially expressed proteins in exosomes from the three groups as described above. **(D, H, L)** List of the top 5 upregulated and downregulated proteins among the differentially expressed proteins from the three groups described above.

Label−free shotgun proteomics was used to quantitatively analyze the exosomal protein samples obtained from the three groups. A total of 1086 proteins at a 1% false discovery rate were both identified through the resulting mass spectra in GO group and GD group (**Figure 2A**). The differentially expressed proteins included 166 upregulated proteins and 6 downregulated proteins in the GO group compared to the GD group. The significantly differential expression of these proteins between these two groups was also shown by the volcano plot and heatmap (**Figures 2B, C**). Among the proteins, KRT13, KRT4, HIST1H1D, APOA-IV and GLUL were the top 5 upregulated proteins, and KRT9, LACRT (fragment), LACRT, APOD, and KRT1 were the top 5 downregulated proteins (**Figure 2D**). 1059 proteins were in common in GD group and healthy controls (**Figure 2E**). Nine upregulated proteins and 95 downregulated proteins in the GD group compared to the healthy control group are presented as a volcano plot and heatmap (**Figures 2F, G**). The most variation in upregulated and downregulated genes is listed in **Figure 2H**. Venn diagram showed that 1070 common proteins between the GO group and healthy controls (**Figure 2I**). The differentially expressed proteins between the GO group and the healthy control group included 28 upregulated and 19 downregulated proteins (**Figures 2J, K**). The top 5 upregulated and downregulated proteins are listed in **Figure 2L**.

### Enrichment analysis of differentially expressed proteins

**Figure 3:**
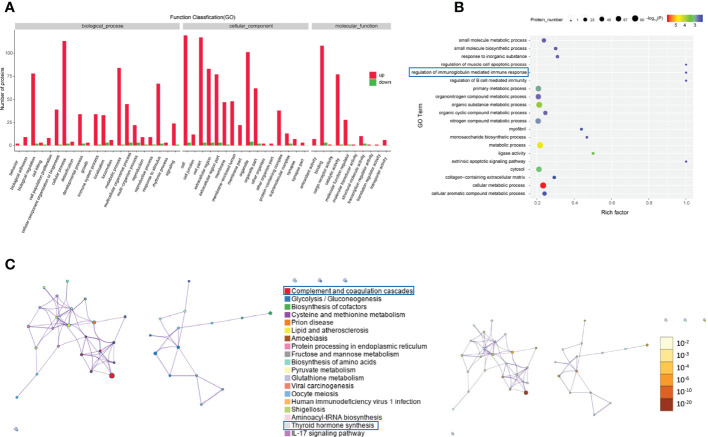
Gene Ontology and KEGG analyses of differentially expressed proteins from the GO group and the GD group. **(A-C)** Gene Ontology annotation **(A)**, Gene Ontology enrichment **(B)**, and KEGG enrichment **(C)** analyses of differentially expressed proteins in exosomes from the GO group and the GD group.

GO analysis was performed to enrich and cluster the differentially expressed proteins between active/severe GO patients and GD patients. In Gene Ontology annotation analysis, we found that the proteins were enriched in several biological processes, including biological regulation, cellular processes, immune system processes, metabolic processes and responses to stimuli. In addition, they were enriched in cellular components such as cell part, extracellular region and organelle. Binding, catalytic activity and molecular function regulator were the Gene Ontology molecular function terms with which the differentially expressed proteins were enriched (**Figure 3A**). In the Gene Ontology enrichment analysis, it was shown that regulation of muscle cell apoptotic process, regulation of the immunoglobulin-mediated immune response and cellular metabolic processes were the main enriched terms associated with these proteins (**Figure 3B**).

The KEGG pathway analysis indicated that complement and coagulation cascades, thyroid hormone synthesis, lipid and atherosclerosis and the IL-17 signaling pathway were the main KEGG pathways of the differentially expressed proteins (**Figure 3C**).

**Figure 4:**
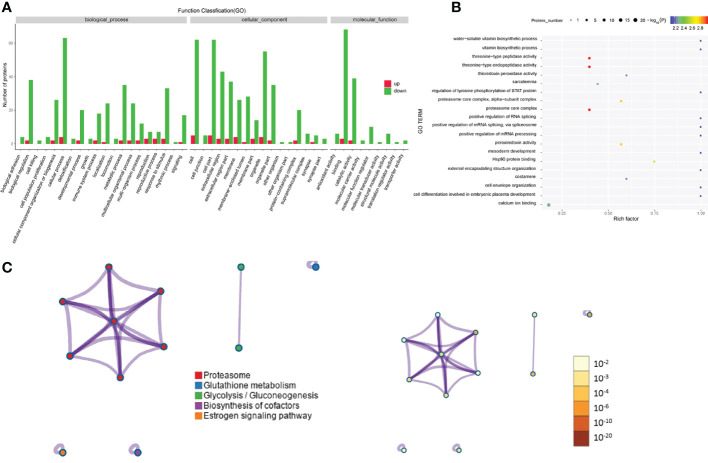
Gene Ontology and KEGG analyses of differentially expressed proteins from the GD group and the healthy control group. **(A-C)** GO annotation **(A)**, GO enrichment **(B)**, and KEGG enrichment **(C)** analysis of differentially expressed proteins in exosomes from the GD group and the healthy control group.

Regarding the differentially expressed proteins in tear exosomes of GD patients and healthy controls, the enriched Gene Ontology biological processes included immune system processes, metabolic processes and cellular processes (**Figure 4A**). Proteins were also enriched in vitamin biosynthetic process and calcium ion binding (**Figure 4B**). Proteasome and biosynthesis of cofactors were the KEGG pathways in which proteins were enriched (**Figure 4C**).

**Figure 5:**
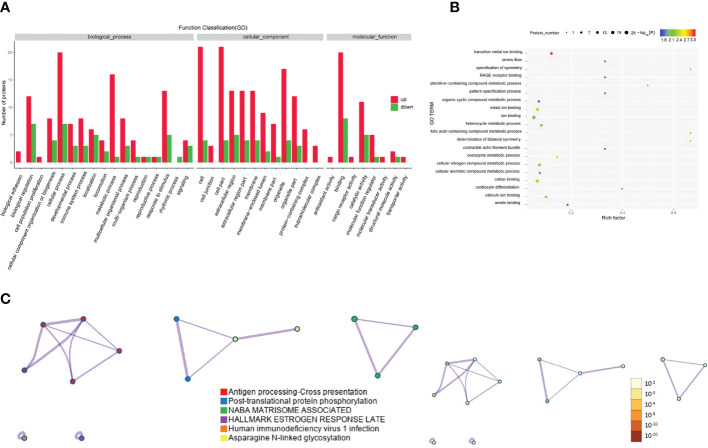
Gene Ontology and KEGG analyses of differentially expressed proteins from the GO group and the healthy control group. **(A-C)** Gene Ontology annotation **(A)**, Gene Ontology enrichment **(B)**, and KEGG enrichment **(C)** analyses of differentially expressed proteins in exosomes from the GD group and healthy controls.

The Gene Ontology annotations and enrichments were analyzed to categorize the differentially expressed proteins between GO patients and healthy controls. Metabolic process, cellular process and response to stimulus were the top Gene Ontology biological processes that were enriched (**Figure 5A**). Proteins were also enriched for the compound metabolic process and ion binding Gene Ontology terms (**Figure 5B**). KEGG pathway analysis indicated that antigen processing-cross presentation and posttranslational protein phosphorylation were the main KEGG pathways related to the differentially expressed proteins (**Figure 5C**).

### Biomarker discovery between the GO group and GD group

**Figure 6:**
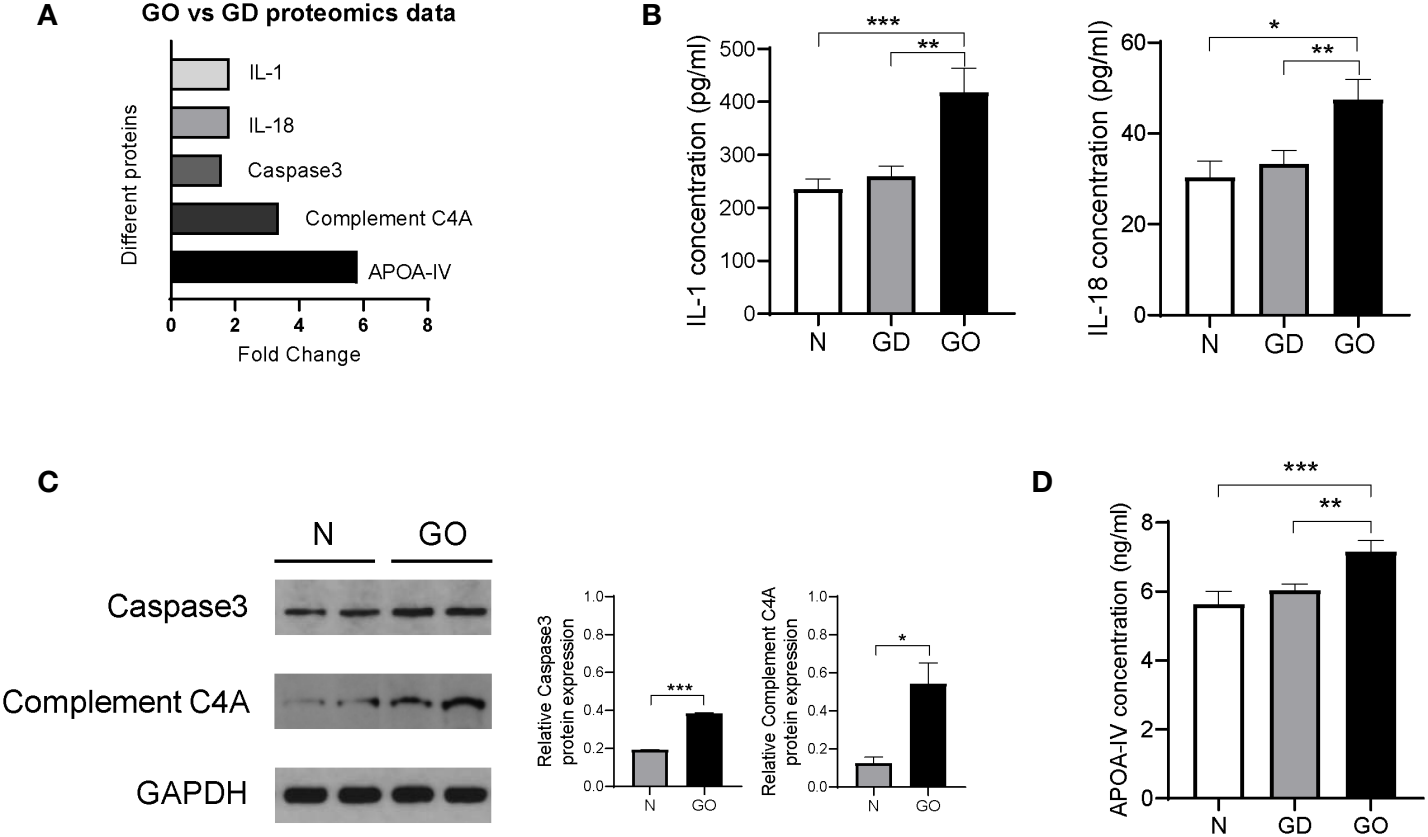
Validation of differentially expressed proteins in exosomes from the GO patients, GD patients and controls. **(A)** Proteins in exosomes from the GO group and the GD group that were significantly varied in the proteomics analysis are shown in columns. **(B)** Inflammatory cytokines such as IL-1 and IL-18 among three groups. **(C)** Representative images and protein expression levels of Caspase-3, Complement C4A and GAPDH in western blots in GO patients and controls. **(D)** Apolipoprotein A-IV concentrations were measured by ELISA in the GO group, the GD group and the healthy control group. * P<0.05, ** P<0.01, *** P<0.001.

Many differentially expressed proteins between the GO group and GD group were found by MS experiments, which showed that the method is an effective and emerging method for protein profiling. Inflammatory factors such as IL-1 (fold change = 1.8, p = 0.03) and IL-18 (fold change = 1.8, p = 0.04) were all upregulated in the GO group compared with the GD group (**Figure 6A**). The conclusion was the same as that of a previous report in which the expression of IL-1 was found to be higher in affected orbital tissues and in orbital fibroblasts from patients with GO than in tissues from unaffected controls (16). IL validation results showed that the levels of IL-1 and IL-18 were increased in GO patients compared with GD patients and healthy controls (**Figure 6B**).

Caspase-3 (fold change = 1.6, p = 0.04) was related to the activation cascade of apoptosis execution caspases, which was upregulated in GO patients compared with GD patients (**Figure 6A**). We used western blot experiments to verify the MS results. Relative Caspase-3 protein expression in retrobulbar adipose tissue of GO patients was 1.9 times that in controls (**Figure 6C**). The pathogenesis of Graves’ ophthalmopathy involves the death of retro-orbital fibroblasts or adipocytes through the extracellular apoptosis pathway and upregulation of Caspase-3 expression (17).

Our results also revealed that complement C4A (fold change = 3.3, p < 0.001) was upregulated in tear exosome proteins of the GO group compared with the GD group (**Figure 6A**). Western blots showed that the expression levels of complement CA4 in retrobulbar adipose tissue of GO patients were 4.3 times those in controls (**Figure 6C**). Complement C4A is a mediator of the local inflammatory process and is essential for the propagation of the classical complement pathway (18, 19). Complement C4A is an inflammation-related protein, and its content varies in different inflammatory diseases, such as GO and GD. Another study has confirmed that complement C4A is markedly upregulated in serum samples of GO patients compared with controls (20).

It has been demonstrated that the main role of HDL-associated APOA-IV is the compartmentalization of molecules to form functional platforms for immunity processes including toll-like receptors (TLRs).TLRs are expressed by many cells including Langerhans cells, keratinocytes and several immune cells (21). Furthermore, TLRs are implicated in the pathogenesis of thyroid associated diseases and GO (22). However, the mechanism between APOA-IV and GO cannot yet be shown and need be explored. The MS results showed that the expression of the lipid metabolism–related protein apolipoprotein A-IV (fold change = 5.8, p = 0.01) was increased significantly in GO patients; this protein carries lipids and act as a ligand for receptors or cofactors of enzymes (23) (**Figure 6A**). Recently, studies have found that apolipoprotein A-IV expression is significantly higher in diabetic patients with dry eye syndrome than in patients with only diabetes, which suggests that apolipoprotein A-IV is important for stabilizing tear films (24). We further verified the results with serum samples from the three groups (**Figure 6D**). The APOA-IV concentration was higher in GO patients than in GD patients and healthy controls and was not different between GD patients and healthy controls. The outcome indicates that APOA-IV is helpful for distinguishing patients with GD and GO.

## Discussion

In this study, we aimed to investigate specific proteins or cytokines in tear exosomes which are differentially expressed in GO patients compared with GD and healthy control. We extracted proteins from tear exosomes and used an LC−MS/MS system to analyze the differentially expressed proteins from active and severe GO patients, GD patients and healthy controls. The number of exosomes was also increased in the GO group, which may have led to enhanced activity, including the release of key inflammatory cytokines to remodel tissue and inflammation. The differentially expressed proteins included 166 upregulated proteins and 6 downregulated proteins in the GO group compared with the GD group.

Since exosomes can be released from almost all cells, lacrimal gland cells and corneal fibroblasts might be the sources of exosomes in tear fluid (25). In this study, we demonstrated that some proteins were upregulated in tear exosomes in the GO group compared with the GD group and the healthy control group. Evidence have demonstrated that the levels of cytokines in tears, including IL-1β, IL-8, and IL-13, are higher in GO patients than in healthy subjects and inactive GO patients, considering their association with CAS (9, 26). In our study, the expression levels of IL-1 and IL-18 in tear exosomes were significantly increased in GO patients, while serum levels were increased markedly.

Inflammatory responses caused by diseases related to the ocular surface can directly damage the epithelial cells of the ocular surface (27). There is evidence that epithelial apoptosis is closely related to the occurrence of dry eye (28, 29), but whether it is involved in the occurrence of GO is unclear. Our results showed that Caspase-3 levels in exosomes from GO patients were higher than those in exosomes from controls, while the levels of Caspase-3 in extraocular muscles in GO patients were significantly higher. These results suggest that long-term exposure to the immune environment leads to apoptosis of ocular epithelial cells through the infiltration and injury of ocular surface inflammatory cells.

Complement component C4 (C4) is hydrolyzed by C1s to form C4a and C4b, which play roles in complement activation, promoting phagocytosis, preventing immune complex deposition and neutralizing viruses (30). The complement system, considered the first line of defense against extrinsic and intrinsic antigens, induces inflammation and direct lysis of antigens. The levels of complement component C4 might determine the intrinsic strength of the classical complement pathway. Yu-Huei Liu et al. demonstrated that the complement component C4 may be able to be developed for clinical application for GD and for vitiligo in GD patients (31). They demonstrated the association between very few copies of C4A, but not C4B, and clinical features of GD, including susceptibility to GO. In our study, the levels of C4A in the tear exosomes and extraocular muscles were increased significantly.

Evidence has shown that lipoproteins, considered lipid transporters, play important roles in many aspects of immunity (32). High-density lipoprotein (HDL) is the most abundant lipoprotein because of its composition and biological function. There are few studies about the role of HDL in thyroid diseases, although HDL has been confirmed to affect the activity of immune cells (33). Apolipoprotein (apo) A-I, ApoA-IV, and apoC-III, and lyso-phosphatidylcholines are the components of HDL. Previous studies have reported that these apolipoproteins can significantly suppress immune responses (34). Roula et al. recently reported that the serum concentrations of ApoA-IV in allergic rhinitis patients are lower than those in controls (33). Interestingly, our studies provide evidence that the levels of apolipoprotein ApoA-IV in tears and serum are increased markedly. HDL composition and function are influenced by the immune environment, further influencing disease progression and the risk of GO disease.

In our study, the limitation is the lacking of comparison between inactive and active GO patients. Whether the exosomes from tear can distinguish the severity and activity of GO progress needs to be studied further. Next, our study will be continued to analyze the exosomes with disease progression and treatment.

In conclusion, the numbers of exosomes and the levels of exosomal proteins are higher in the tear fluids of GO patients than in those of GD patients and controls. Specific proteins, such as Caspase-3, C4A and APOA-IV, show elevated expression in exosomes from GO patients, implying that they may play important roles in GO pathogenesis.

## Data availability statement

The raw data supporting the conclusions of this article will be made available by the authors, without undue reservation.

## Ethics statement

The studies involving human participants were reviewed and approved by No. TRECKY2016-003. The patients/participants provided their written informed consent to participate in this study.

## Author contributions

JY and DL conceived and designed the study. TS and RZ designed and performed the experiments, and wrote the draft of the manuscript. All authors contributed to the article and approved the submitted version.
